# Feeding, mating and animal wellbeing: new insights from phylogenetic comparative methods

**DOI:** 10.1098/rspb.2022.2571

**Published:** 2023-03-08

**Authors:** Emma L. Mellor, Georgia J. Mason

**Affiliations:** ^1^ Bristol Veterinary School, University of Bristol, Bristol, UK; ^2^ Department of Integrative Biology, University of Guelph, Guelph, Canada

**Keywords:** animal welfare, conservation, zoo, behavioural needs, comparative method

## 1. Introduction

Some species tend to thrive in captivity, while others risk health and reproductive problems. This enables the use of phylogenetic comparative methods (PCMs) to identify aspects of natural biology that predispose species to faring poorly or well. Risk factors can then suggest new ways to improve animal care. A steady trickle of studies has applied PCMs to animal welfare over the last two decades, Lewis *et al*. [1] providing the latest. Here we contextualize this new work, and suggest further research it might inspire.

Lewis *et al*. [1] conducted a meta-analysis of the prevalence of ‘stereotypic behaviour’ (SB), an ethological welfare indicator in which sub-optimally kept animals perform repetitive actions reflecting frustration, repeated attempts to cope, or neurological impairment (cf. [2–4]). Ungulates (e.g. zoo-housed giraffes [*Giraffa camelopardalis*], stabled horses [*Equus caballus*] and farmed pigs [*Sus scrofa*]) are prone to oral SBs, like bar-chewing and tongue-rolling, suggested to derive from vacuum or redirected foraging triggered by artificial diets [4]. Lewis *et al*. [1] created an impressive literature-derived database for 38 ungulate species and ran ground-breaking phylogenetically informed multi-level Bayesian regression models: instead of only using species summary statistics as datapoints (the norm), they also incorporated data on individuals' husbandry (e.g. enclosure size, feeding regimes). Both species and husbandry effects supported the foraging hypothesis. SB was more prevalent in naturally browsing species (those primarily eating non-grass plants like tree leaves) than species naturally reliant on grazing; and in individuals fed ‘concentrates’ (e.g. processed pellets), or discrete meals rather than *ad libitum*. Two other potential risk factors were also explored: species-typical ranging behaviour or activity levels (with no discernible effects), plus social organization. Social housing regimes had no effects; but naturally promiscuous species had more prevalent SB: an intriguing yet puzzling pattern discussed below.

These findings reveal similarities and differences compared to other groups (Table 1; reviewed by [11]). For example, that natural ranging behaviour seems to have no welfare significance for Ungulata contrasts with Carnivora and Primates. This could explain why ungulates seldom perform route-tracing SBs (e.g. pacing), and suggests underlying differences in ranging behaviour's plasticity and/or reward value. By contrast, ungulates resemble parrots, whose self-directed feather-chewing and -plucking is similarly predicted by foraging niche [3]. This suggests three potential similarities between these otherwise divergent groups. First, foraging is naturally time-consuming for both, and accessing food requires extensive oral manipulation (e.g. navigating thorns and branches with lips; tearing and crushing with bills). This could lead to foraging behaviours evolving into intrinsically rewarding ‘behavioural needs’ [3,4]. Furthermore, these animals' wild food items are naturally patchy, potentially driving the evolution of motivational systems wherein ingestion triggers intense local search: a functionless response if food is provided in feeders [4]. Third, nutritional and/or physical differences between wild and captive diets could instead be the key (e.g. captive browsers are often fed grass or lucerne hay, not a natural diet [6]). If such proxies are inadequate, this could trigger deficit-led oral searching behaviours, induce coping responses (e.g. the ingestion of saliva or other items), or even compromise gastrointestinal health [3,4]. These three, non-mutually exclusive hypotheses could now be tested in both disparate, yet seemingly convergent, taxa.

**Table 1.** Welfare risk factors identified by phylogenetic comparative methods (PCMs): Summarized results from the eight studies investigating aspects of behavioural biology as welfare risk factors in captivity. Ticks indicate risk factors; crosses, rejected potential risk factors; and question marks, untested hypotheses. SB = stereotypic behaviour.

potential risk factor	carnivores [2,5]: route-tracing SBs	ungulates [1,6,7]: SB; captivity effects on ageing/survival (A, S)^b^	Primates [8]: route-tracing (RT) and hair-plucking (HP) SBs	Psittaciformes [3,9]: whole-body (WB), feather-damaging (FD), and other oral (O) SBs
*relating to movement in the wild*				
extensive ranging/travel	✓	✗	✓_RT_	✗
*relating to foraging*				
diet type	✗^a^	✓_SB, A, S_	?	✗
extensive food handling	?	?	?	✓_FD_
*relating to social/sexual biology*				
large natural group size	?	✗	✓_HP_	✗
mating system	?	✓_SB, A_	?	?
*relating to intelligence*				
large relative brain sizes	✗	?	?	✓_WB, O_

^a^Kroshko *et al*. [2] did find one foraging-related effect, linking route-tracing with long chase distances. However, this only stemmed from five species; no other foraging variable was predictive; and it was not subsequently replicated [10].

^b^Note Müller *et al*.'s [6] risk factors were sex-specific: for females being a browser (versus grazer) and for males being polygamous (versus monogamous; see text).

Lewis *et al*.'s [1] findings also show parallels with previous PCM research on zoo-housed ruminants [6,7]. Using a metric that controls for intrinsic longevity, Müller *et al*. [6] revealed that female zoo-housed browsers have relatively shortened lifespans, typically living smaller proportions of their species' maximum lifespans compared to grazers. For instance, zoo-housed female moose (*Alces alces*) and giraffe, both browsers, on average live 27% and 36% of their respective maximum lifespans; whereas the grass-eating Arabian oryx (*Oryx leucoryx*) and black wildebeest (*Connochaetes gnou*) have longer lifespans in zoos (on average living until 59% and 52% of their species’ maxima [6]). Lemaître *et al*. [7] similarly found that, while actuarial senescence rates of zoo-housed ruminants are lower than their wild-living counterparts, this benefit is markedly reduced for naturally browsing species.

Together, this indicates that captive browsers risk both health and behavioural problems (cf. [4]). We therefore ran new analyses to explore how these inter-relate. These are preliminary, relying on publicly available data from limited sample sizes. But we hope they encourage zoo researchers to test these hypotheses more rigorously by accessing zoo records. Our analyses also demonstrate a valuable tool for identifying extreme datapoints with undue leverage: the ‘influ_phylm’ function (sensiPhy R package [12]). This systematically identifies ‘influential’ species: those whose removal changes focal predictors' estimates by >2 standardized differences. Whether these created artefactual patterns, or masked underlying relationships, can then be assessed. This is an advance over making untested assumptions about certain species. Thus, Lewis *et al*. [1] re-ran models after excluding intensively farmed populations of pigs, cows (*Bos taurus*) and sheep (*Ovis aries*). The concern that these could skew results was sensible, but the authors somewhat arbitrarily did not exclude intensively stabled horses (nor, for zoo-housed animals, ‘studbook’-managed species that might receive extra care; [6]). The tool we use makes such exclusion decisions more objective and transparent.

**Figure 1.**
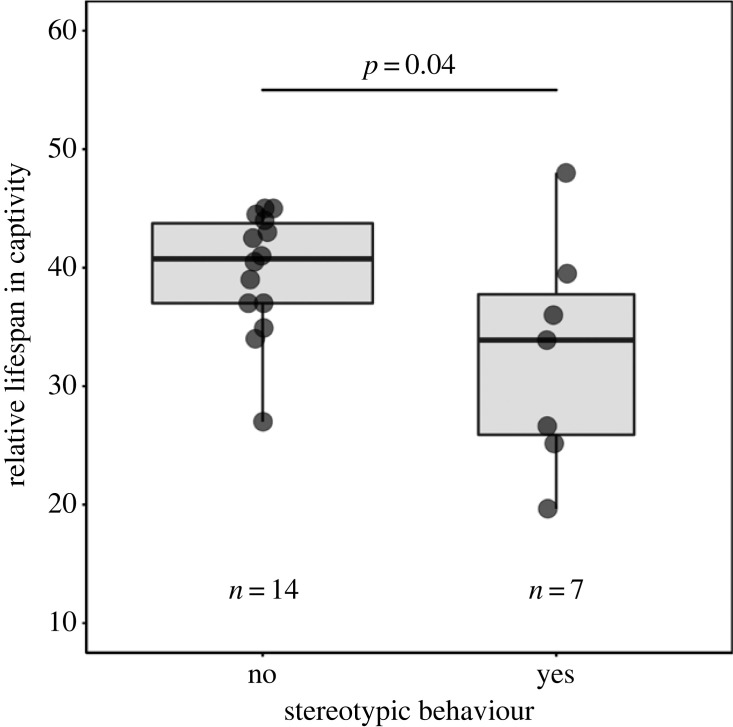
Stereotypic ungulates have relatively shortened lifespans in zoos. To meet model assumptions, we categorized Lewis *et al.*'s data [1], scoring species for reported presence/absence of stereotypic behaviour. For relative lifespans in captivity, we took averages of sex-specific relative lifespans for 16 ruminant species (from [6]), and calculated values for five non-ruminants (from [11]). Analyses used an ultrametric consensus ungulate tree using 1000 trees (from [12]) and phylogenetic generalized least squares regressions in R (cf. [3]). However, to maximize species numbers, we did not follow our preferred rule of excluding species represented by under five individuals [2,3]. Results are, therefore, provisional until replicated with larger datasets. Data points represent species, thick horizontal lines indicate medians, lower and upper hinges the first and third quartiles, and whiskers 1.5 times the interquartile range from the lower and upper quartiles. Stereotypic species (*n* = 7) have shorter captive relative lifespans than non-stereotypic ones (*n* = 14) (*F* = 4.67, d.f. = 19, *p* = 0.04; coef. = −0.07 [−0.13, −0.01], *t* = −2.16, *n* = 21, adjusted *R*^2^ = 0.16, *λ* = 0, *p* = 0.04). The ‘influ_phylm’ function (in [9]) identified three influential species, but after removing each, stereotypic species still had significantly shorter relative lifespans (*p* < 0.0001).

First, we assessed whether SB-prone ungulate species have shortened captive lifespans. This hypothesis appeared supported: a finding not driven by any particular species (indeed removing ‘influential’ datapoints actually strengthened effects; Figure 1). Furthermore, influential species were not intensively farmed (being the sitatunga [*Tragelaphus spekii*], white rhinoceros [*Ceratotherium simum*] and black rhinoceros [*Diceros bicornis*]). So, despite including SB values from intensively farmed cows and sheep, these animals were not model outliers (perhaps reflecting these domesticates' relative adaptation to intensive housing). Future studies should now re-investigate this intriguing apparent SB-survivorship link, using larger sample sizes (of both individuals and species; cf. [1,11]), and perhaps actuarial senescence data (e.g. [7]) to test whether SB-prone species have accelerated ageing in captivity.

We also attempted to investigate whether captive browsers are more prone than grazers to digestive ill-health, which could cause both their elevated SB [4] and shortened captive lifespans [6]. We used necropsy report data for 30 ruminant species in a South African zoo, on four diet-related clinical signs: parakeratosis (hardened, enlarged ruminal papillae), rumenitis and/or ruminal acidosis (PRA); abnormal stomach contents (e.g. foreign objects); abnormal tooth-wear; and poor body condition [15]. Analyses yielded preliminary evidence that naturally browsing species could be more vulnerable to PRA, abnormal tooth wear and poor body condition (electronic supplementary material, table S1). However, all three apparent patterns relied on excluding different influential species, perhaps suggesting stochastic effects driven by sampling error (there being few individuals per species). We therefore hope researchers with access to multi-zoo veterinary records will test this hypothesis more robustly.

Together, although requiring more research, these findings highlight the vulnerability of naturally browsing ungulates to health and welfare problems in captivity: ethically concerning if unresolved, and practically concerning if they reduce chances of forming self-sustaining captive populations and/or subject animals to intense new selection pressures. Reciprocally, however, they also emphasize that grazers seem pre-adapted to thrive in human care. Perhaps this is why many long-domesticated ungulates are grazers (e.g. sheep, cattle, horses), and why captive breeding and reintroduction has proved so successful for the Arabian oryx, Père David's deer (*Elaphurus davidianus*) and Przewalski's horse (*Equus ferus*) (cf. www.iucnredlist.org).

Like Lewis *et al*. [1], Müller *et al*. [6] also found a mating system effect: compared with monogamous species, zoo-housed male polygamous ruminants have relatively shortened lifespans. Despite using survival metrics that controlled for intrinsic effects, they suggested this pattern reflects intrinsic costs of male reproductive biology. Lewis and colleagues instead interpreted their own findings as reflecting welfare harms from frustrated access to mates. However, they included no monogamous species (unlike [6]), and missing cases precluded assessing whether their apparent mating system effect actually reflected foraging niche [1]. Interpreting and reconciling these two studies is therefore tricky. Nevertheless, both imply that species' evolved mating systems might influence their captive wellbeing: a broad idea to date little considered in welfare research, despite the crucial role of mating and mate choice in fitness (suggesting the evolution of strong underlying motivations for relevant behaviours); the tight captive management of mating and mate choice by humans; and experimental studies indicating that constraining these can harm welfare [16–18]. We therefore propose three new hypotheses, each testable using statistical approaches that can incorporate both enclosure-level variation in husbandry and species differences (cf. [1]), along with sex too (since predictions may differ between males and females). The first hypothesis is that having naturalistic opportunities to select (or avoid) particular mates is important for welfare. This predicts that in captive conditions preventing mate choice, welfare will be reduced in species that are naturally choosy about mates. The second is that having naturalistic numbers of copulatory opportunities is important for welfare. This predicts that in captive conditions which prevent mating, welfare will be reduced in species that naturally mate often. Finally, the third is that having naturalistic numbers of partners is important for welfare. This predicts that in captive conditions which limit the number of potential mates, welfare will be reduced in species that naturally have multiple partners. Together, this illustrates how PCMs can both *generate* novel hypotheses and be valuable to *test* such hypotheses. It also highlights the value of Lewis *et al*. [1]'s new way to simultaneously investigate effects of species traits and husbandry (and potentially other within-species factors too).

These new ideas, and the question marks in Table 1, thus represent hypotheses that could yet be tested by comparing welfare across species. We hope that more researchers will apply this still under-used approach to animal welfare because it has enormous value. It can help efficiently improve captive husbandry, by identifying which aspects of natural lifestyles need mimicking to maximize wellbeing (e.g. the ticks in Table 1) and which seem *not* to be important (e.g. the crosses in Table 1). It has fundamental value too, raising new ideas about the adaptive design of motivational systems. Furthermore, PCMs can identify the types of species most prone to poor captive welfare and, reciprocally, those inherently easy to keep happy and healthy. And understanding *these* is also relevant beyond welfare, potentially generating new insights into historic animal domestications and contemporary conservation successes.

## Data Availability

Data and code to support the analyses and a figure provided here can be found at Dryad Digital Repository: (http://dx.doi.org/10.5061/dryad.v6wwpzh0g) [19]. The data are provided in electronic supplementary material [20].
