# Correction to: ‘Feeding, mating and animal wellbeing: new insights from phylogenetic comparative methods’

**DOI:** 10.1098/rspb.2023.0750

**Published:** 2023-05-10

**Authors:** Emma L. Mellor, Georgia J. Mason

*Proc. R. Soc. B* **290**, 20222571. (Published online 1 March 2023). (https://doi.org/10.1098/rspb.2022.2571)

This file contains a corrected version of figure 1 featured in our published paper ‘Feeding, mating and animal wellbeing: new insights from phylogenetic comparative methods’. After publication, we discovered a coding error in the calculation of the error bars of our original figure. With unequal sample sizes between the levels of our predictor (i.e. *n* = 14 and *n* = 7; see figure 1), in this correction, we present the raw data as box-and-whisker plots. We also have made minor edits to figure 1’s caption to describe what the box-and-whisker plots show. The original statistical output is not affected, and so our original results and conclusions are unchanged.

**Figure 1.**
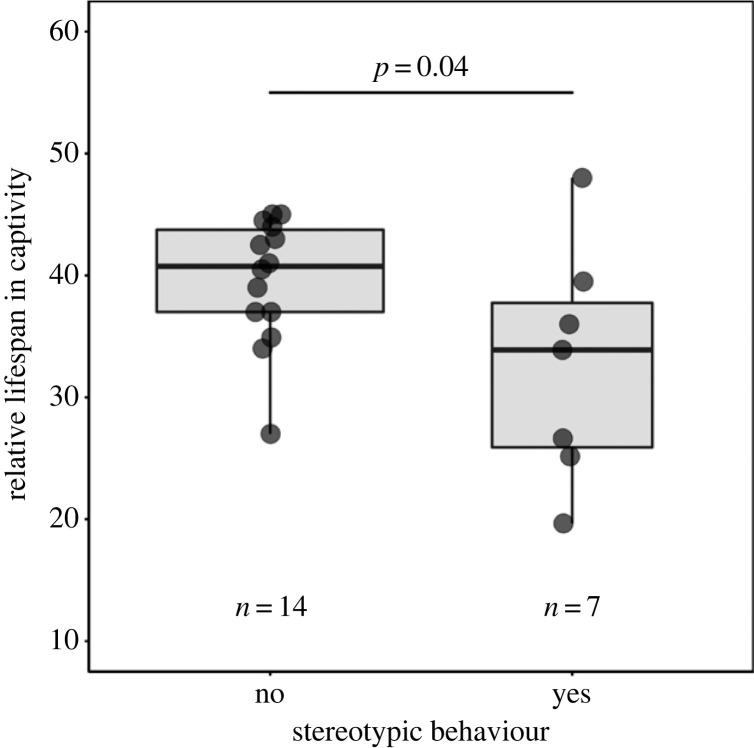
Stereotypic ungulates have relatively shortened lifespans in zoos. To meet model assumptions, we categorized Lewis *et al.*'s data [1], scoring species for reported presence/absence of stereotypic behaviour. For relative lifespans in captivity, we took averages of sex-specific relative lifespans for 16 ruminant species (from [6]), and calculated values for five non-ruminants (from [11]). Analyses used an ultrametric consensus ungulate tree using 1000 trees (from [12]) and phylogenetic generalized least squares regressions in R (cf. [3]). However, to maximize species numbers, we did not follow our preferred rule of excluding species represented by under five individuals [2,3]. Results are, therefore, provisional until replicated with larger datasets. Data points represent species, thick horizontal lines indicate medians, lower and upper hinges the first and third quartiles, and whiskers 1.5 times the interquartile range from the lower and upper quartiles. Stereotypic species (*n* = 7) have shorter captive relative lifespans than non-stereotypic ones (*n* = 14) (*F* = 4.67, d.f. = 19, *p* = 0.04; coef. = −0.07 [−0.13, −0.01], *t* = −2.16, *n* = 21, adjusted *R*^2^ = 0.16, *λ* = 0, *p* = 0.04). The ‘influ_phylm’ function (in [9]) identified three influential species, but after removing each, stereotypic species still had significantly shorter relative lifespans (*p* < 0.0001).

This has been corrected on the publisher's website.

